# A retrospective comparative study of tenofovir alafenamide and tenofovir disoproxil fumarate in chronic hepatitis B patients: Renal and bone safety versus antiviral efficacy

**DOI:** 10.1097/MD.0000000000047350

**Published:** 2026-01-30

**Authors:** Lizhi Wang, Guotao Pi, Dan Lu, Yan Wang, Wenjin Sun

**Affiliations:** a Infection Department, Ezhou Central Hospital, Ezhou, Hubei Province, China; b Digestive Medicine Department, Ezhou Central Hospital, Ezhou, Hubei Province, China.

**Keywords:** antiviral efficacy, bone mineral density, chronic hepatitis B, eGFR, renal safety, tenofovir alafenamide, tenofovir disoproxil fumarate

## Abstract

Long-term nucleos(t)ide analogue (NA) therapy is required for chronic hepatitis B (CHB), and drug-related renal and bone toxicities remain a major clinical concern. The real-world differences in renal and bone safety between tenofovir alafenamide (TAF) and tenofovir disoproxil fumarate (TDF), as well as their relationship with antiviral efficacy, need further validation in local populations. We conducted a single-center retrospective cohort study enrolling 102 adult CHB patients (TAF, n = 52; TDF, n = 50) who initiated therapy within the past 3 years and were followed up for 6 and 12 months. The primary outcomes were changes in estimated glomerular filtration rate (ΔeGFR, mL/min/1.73 m²) and lumbar spine bone mineral density (% change). Secondary outcomes included undetectable hepatitis B virus DNA rate, alanine aminotransferase normalization, and hepatitis B e antigen seroconversion. Safety endpoints comprised hypophosphatemia, proteinuria, acute kidney injury, grade ≥ 3 adverse events, and treatment discontinuation. Group comparisons were performed using Welch’s *t* test or *χ*²/Fisher’s exact test, and sensitivity analyses were conducted using propensity score matching. Baseline characteristics were comparable between groups after propensity score matching. At 6 months, changes in estimated glomerular filtration rate did not differ significantly (0.78 ± 4.91 vs −0.39 ± 5.00; *P* = .236), but at 12 months, TAF showed superior renal preservation (1.22 ± 5.81 vs −1.58 ± 5.68; *P* = .016). Lumbar bone mineral density changes were similar at 6 months (0.27 ± 1.43% vs −0.20 ± 1.53%; *P* = .113) but favored TAF at 12 months (0.56 ± 1.75% vs −0.47 ± 1.97%; *P* = .006). Virological outcomes, including hepatitis B virus DNA suppression and hepatitis B e antigen seroconversion, were comparable at both time points. Alanine aminotransferase normalization showed a nonsignificant trend favoring TAF at 12 months (73.08% vs 88.00%; *P* = .058). TAF was associated with markedly lower rates of hypophosphatemia (1.92% vs 28.00%; *P* < .001) and less proteinuria at 6 months (3.85% vs 16.00%; *P* = .039). In this retrospective cohort, TAF demonstrated significantly better renal and bone safety profiles than TDF after 12 months of treatment, while achieving equivalent antiviral efficacy. For CHB patients at risk of renal or bone impairment, TAF may represent a safer long-term therapeutic option.

## 1. Introduction

Chronic hepatitis B (CHB) usually requires long-term or even lifelong nucleos(t)ide analogue (NA) therapy to achieve persistent viral suppression and reduce the risk of adverse outcomes such as cirrhosis and hepatocellular carcinoma. Current international guidelines, including those from the American Association for the Study of Liver Diseases and the European Association for the Study of the Liver, recommend first-line agents with a high genetic barrier to resistance, such as entecavir, tenofovir disoproxil fumarate (TDF), and tenofovir alafenamide (TAF). These guidelines also emphasize the importance of regular monitoring of renal function and bone metabolism during long-term therapy. For patients with preexisting renal or bone risk factors – or those who develop related toxicities – TAF-based regimens or switching from TDF to TAF are preferred due to their more favorable safety profiles.^[1,2]^

Long-term exposure to TDF has been associated with proximal renal tubular injury, reductions in estimated glomerular filtration rate (eGFR), and decreased bone mineral density (BMD). TAF, a novel prodrug of tenofovir, achieves equivalent antiviral efficacy at substantially lower plasma tenofovir levels – approximately 90% lower than TDF – thereby reducing systemic exposure and the risk of renal and bone toxicity at the pharmacokinetic level.^[3]^ Two pivotal phase III randomized, double-blind, non-inferiority trials in both hepatitis B e antigen (HBeAg)-positive and HBeAg-negative chronic hepatitis B (CHB) populations confirmed that TAF is non-inferior to TDF in virologic efficacy while demonstrating superior renal and bone safety, with benefits persisting over time.^[4,5]^

Real-world studies have further shown that patients who switch from TDF to TAF experience improvement or stabilization in renal and bone parameters while maintaining virological suppression. However, quantitative differences in outcomes vary across centers and follow-up intervals, suggesting the need for additional localized evidence.^[6,7]^ Recent evidence also indicates potential metabolic trade-offs of TAF, including increases in lipid parameters and body weight in some patients. These considerations underscore the importance of balancing renal and bone safety advantages with appropriate metabolic monitoring.

Based on this background, we conducted a single-center retrospective cohort study to directly compare TAF and TDF with respect to renal and bone safety (ΔeGFR and BMD) and antiviral and biochemical efficacy (hepatitis B virus [HBV] DNA undetectability, alanine aminotransferase (ALT) normalization, and HBeAg seroconversion) over 6- and 12-month follow-up intervals. Propensity score matching (PSM) and sensitivity analyses were applied to enhance robustness. We hypothesized that TAF would offer superior renal and bone safety compared with TDF while maintaining equivalent antiviral efficacy across common clinical subgroups.

## 2. Materials and methods

### 2.1. Study design and reporting standards

This study was approved by the Ethics Committee of Ezhou Central Hospital. This was a single-center, retrospective cohort study conducted in accordance with the STROBE guidelines for observational research. The study included adult patients with CHB who initiated tenofovir-based antiviral therapy for the first time within the past 3 years. Follow-up assessments were prespecified at 6 and 12 months after treatment initiation. The study protocol was approved by the institutional ethics committee, and informed consent was waived because of the retrospective and minimal-risk design. All analyses were performed in compliance with the Declaration of Helsinki.

### 2.2. Study population

Inclusion criteria were as follows: age ≥ 18 years; confirmed CHB without prior exposure to tenofovir alafenamide (TAF) or TDF, or patients restarting therapy meeting the definition of “new initiation”; treatment with TAF (25 mg once daily) or TDF (300 mg once daily) as first-line nucleos(t)ide analogue (NA) monotherapy; availability of key baseline laboratory and imaging data – serum creatinine, ALT, and HBV DNA – obtained within 4 weeks before treatment; and completion of at least 1 follow-up assessment (at 6 or 12 months) for the primary endpoint.

Exclusion criteria included: Severe renal disease, such as dialysis, kidney transplantation, or nephrotic syndrome; pregnancy or lactation; concurrent diseases or medications that significantly affect bone metabolism (e.g., chronic corticosteroid therapy or anti-osteoporotic agents) that could not be adjusted; decompensated cirrhosis requiring urgent intervention; and incomplete key variables or missing data precluding evaluation of the primary endpoint. For patients with partial data, available outcomes were analyzed separately by time window.

### 2.3. Outcomes

#### 2.3.1. Primary outcomes

Renal function change: the primary renal outcome was the change in estimated glomerular filtration rate (ΔeGFR) from baseline to 6 and 12 months. eGFR was calculated using the chronic kidney disease epidemiology collaboration equation based on serum creatinine, which was measured on a standardized platform under regular quality control.

BMD: Lumbar spine (L1–L4) BMD change (%) from baseline was assessed using the same dual-energy X-ray absorptiometry (DXA) equipment at both time points, following manufacturer calibration and international quality standards.

#### 2.3.2. Secondary outcomes

Secondary outcomes included: undetectable HBV DNA rate (defined by the lower limit of quantification of 10–20 IU/mL on the local assay); ALT normalization rate (according to the institutional reference range); and HBeAg seroconversion among patients who were HBeAg-positive at baseline.

#### 2.3.3. Safety outcomes

Safety endpoints were: hypophosphatemia, defined as serum phosphate <0.80 mmol/L; new or worsening proteinuria, based on urine dipstick or quantitative assessment (albumin-to-creatinine ratio or 24-hour protein) exceeding prespecified thresholds; acute kidney injury, defined according to kidney disease: improving global outcomes criteria; and grade ≥ 3 adverse events (AEs) and treatment discontinuation due to AEs. Incidence was calculated cumulatively for both 0 to 6 and 0 to 12 month intervals.

### 2.4. Covariates and data collection

Covariates included age, sex, body mass index, HBeAg status, baseline eGFR, HBV DNA, and ALT levels. All data were retrieved from the hospital information system and laboratory/radiology databases using a standardized electronic data abstraction form. Logical verification and range checks were performed to ensure data accuracy and consistency.

### 2.5. Handling of missing data

The overall rate of missing key variables was low. Analyses were conducted on complete cases. If an endpoint was missing for a specific time point, analyses were based on available data for that outcome window. For rare categorical events (e.g., 0 counts), Fisher’s exact test was preferentially used to ensure statistical robustness.

### 2.6. Statistical analysis

Continuous variables were summarized as mean ± standard deviation and compared using Welch’s *t* test. Categorical variables were summarized as number (percentage) and compared using Pearson’s *χ*² test, or Fisher’s exact test if the expected frequency was <5. For the primary outcomes (ΔeGFR and lumbar BMD change), between-group differences, 95% confidence intervals (CIs), *t* values, and *P*-values were reported at 6 and 12 months.

For secondary and safety outcomes, rates, *χ*²/Fisher *P*-values, odds ratios, and risk differences (95% CIs) were presented. Time trends were visualized using estimated marginal means with 95% CIs, and Kaplan–Meier analysis (log-rank test) was used to assess time-to-target events, optionally supplemented by Cox proportional hazard ratios.

To minimize confounding, PSM was performed using 1:1 nearest-neighbor matching without replacement and a caliper width of 0.2 × standard deviation of the logit score. Covariate balance was evaluated by standardized mean difference (SMD < 0.20). All primary comparisons were repeated in the matched cohort as sensitivity analyses, and Fisher’s test was applied for rare events.

## 3. Results

### 3.1. Study population and baseline characteristics

A total of 102 patients with CHB were included, comprising 52 in the TAF group and 50 in the TDF group. Baseline demographic and clinical characteristics were generally comparable between the 2 groups, with no statistically significant differences in age, sex, body mass index, HBeAg positivity, baseline eGFR, HBV DNA, or ALT levels (all *P* > .05; Table 1). After 1:1 PSM, covariate balance was achieved (standardized mean difference < 0.20 for most variables), confirming good comparability between matched cohorts (Table 2).

**Table 1:** Baseline characteristics of the original cohort.

Variable	TAF (n = 52)	TDF (n = 50)	*t*/*χ*²	*P*
Age, yr	44.36 ± 10.91	44.54 ± 9.49	−0.088	.930
Male, n (%)	35 (67.31%)	35 (70.00%)	0.086	.770
BMI, kg/m²	24.05 ± 3.10	23.54 ± 4.31	0.677	.500
HBeAg positive, n (%)	27 (51.92%)	32 (64.00%)	1.525	.217
Baseline eGFR, mL/min/1.73 m²	99.79 ± 13.72	99.69 ± 15.40	0.036	.971
Baseline HBV DNA, log_10_ IU/mL	6.13 ± 0.82	6.02 ± 0.74	0.672	.503
Baseline ALT, U/L	61.07 ± 31.24	55.66 ± 27.60	0.928	.356

ALT = alanine aminotransferase, BMI = body mass index, eGFR = estimated glomerular filtration rate, HBeAg = hepatitis B e antigen, TAF = tenofovir alafenamide, TDF = tenofovir disoproxil fumarate.

**Table 2:** Baseline characteristics after PSM.

Variable	TAF matched (n = 50)	TDF matched (n = 50)	SMD
Age, yr	44.30 ± 11.08	44.54 ± 9.49	0.023
Male, n (%)	34 (68.00%)	35 (70.00%)	0.043
BMI, kg/m²	24.11 ± 3.00	23.54 ± 4.31	0.153
HBeAg positive, n (%)	27 (54.00%)	32 (64.00%)	0.204
Baseline eGFR, mL/min/1.73 m²	99.33 ± 13.74	99.69 ± 15.40	0.025
Baseline HBV DNA, log_10_ IU/mL	6.14 ± 0.83	6.02 ± 0.74	0.146
Baseline ALT, U/L	60.31 ± 31.01	55.66 ± 27.60	0.159

ALT = alanine aminotransferase, BMI = body mass index, eGFR = estimated glomerular filtration rate, HBeAg = hepatitis B e antigen, SMD = standardized mean difference, TAF = tenofovir alafenamide, TDF = tenofovir disoproxil fumarate.

### 3.2. Primary outcomes

#### 3.2.1. Renal function

**Figure 1.**
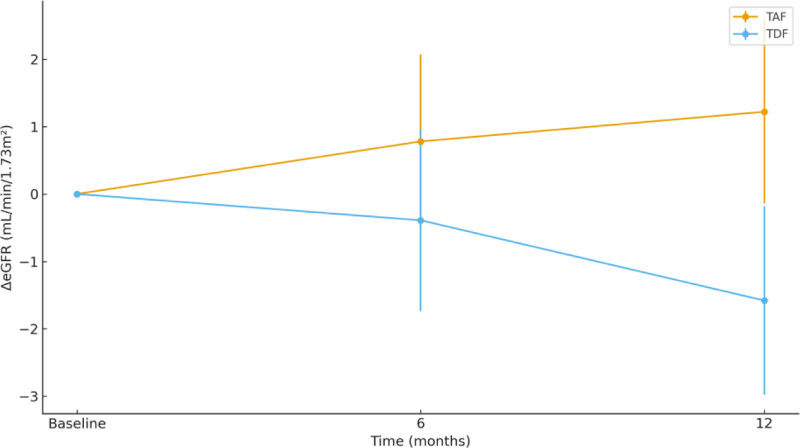
Estimated marginal means of ΔeGFR over time (TAF vs TDF). eGFR = estimated glomerular filtration rate, TAF = tenofovir alafenamide, TDF = tenofovir disoproxil fumarate.

No significant between-group difference in ΔeGFR was observed at 6 months (0.78 ± 4.91 vs −0.39 ± 5.00 mL/min/1.73 m²; *P* = .236). However, at 12 months, TAF showed significantly better renal preservation than TDF (1.22 ± 5.81 vs −1.58 ± 5.68 mL/min/1.73 m²; *P* = .016). The estimated marginal mean trajectory demonstrated a progressive divergence between groups starting at 6 months (Fig. 1; Table 3).

**Table 3:** Primary and secondary outcomes at 6 and 12 months.

Outcome	TAF (n = 52)	TDF (n = 50)	*t*/*χ*²	*P*
ΔeGFR 0–6 mo, mL/min/1.73 m²	0.78 ± 4.91	−0.39 ± 5.00	1.191	.236
ΔeGFR 0–12 mo, mL/min/1.73 m²	1.22 ± 5.81	−1.58 ± 5.68	2.457	.016
Lumbar spine BMD change 0–6 mo, %	0.27 ± 1.43	−0.20 ± 1.53	1.599	.113
Lumbar spine BMD change 0–12 mo, %	0.56 ± 1.75	−0.47 ± 1.97	2.796	.006
HBV DNA undetectable 6 mo, n (%)	47 (90.38%)	44 (88.00%)	0.151	.698
HBV DNA undetectable 12 mo, n (%)	48 (92.31%)	48 (96.00%)	0.628	.428
ALT normalization 6 mo, n (%)	30 (57.69%)	36 (72.00%)	2.285	.131
ALT normalization 12 mo, n (%)	38 (73.08%)	44 (88.00%)	3.601	.058

ALT = alanine aminotransferase, BMD = bone mineral density, eGFR = estimated glomerular filtration rate, TAF = tenofovir alafenamide, TDF = tenofovir disoproxil fumarate.

#### 3.2.2. Bone mineral density (BMD)

**Figure 2.**
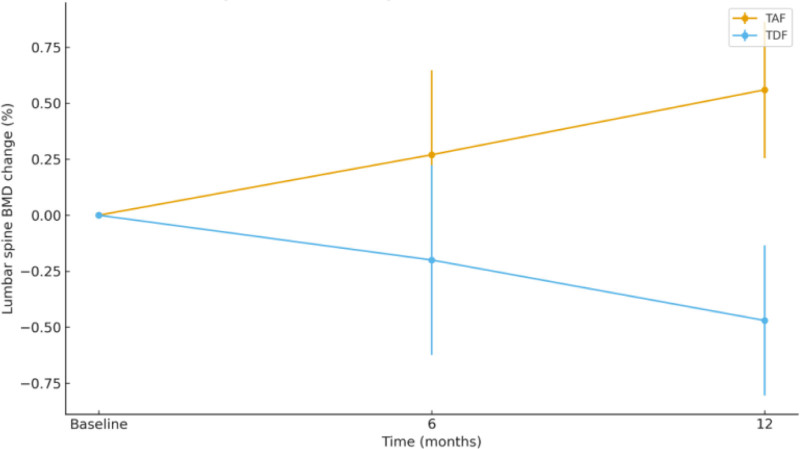
Lumbar spine BMD (%) change over time by treatment group. BMD = bone mineral density.

Lumbar spine BMD changes were not significantly different between groups at 6 months (0.27 ± 1.43% vs −0.20 ± 1.53%; *P* = .113). At 12 months, however, the TAF group showed a significantly greater increase in BMD compared with the TDF group (0.56 ± 1.75% vs −0.47 ± 1.97%; *P* = .006), indicating improved bone safety with TAF (Fig. 2; Table 3).

### 3.3. Secondary outcomes

#### 3.3.1. Virological response

**Figure 3.**
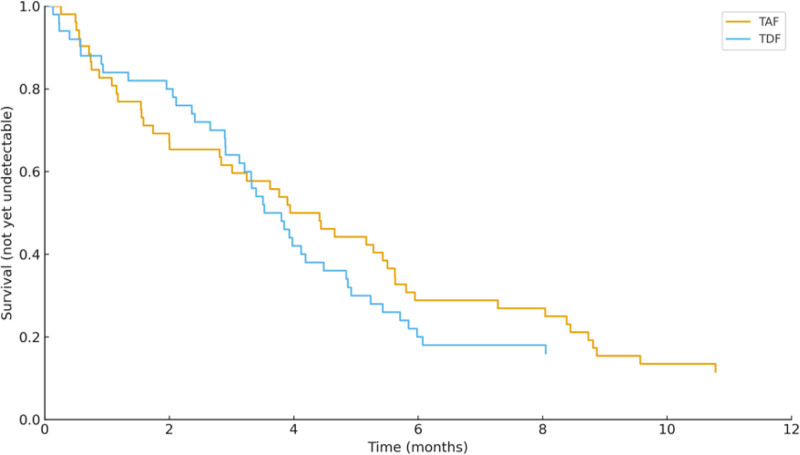
Kaplan–Meier curves for time to HBV DNA undetectable. HBV = hepatitis B virus.

HBV DNA suppression rates were comparable between groups at both 6 months (90.38% vs 88.00%; *P* = .698) and 12 months (92.31% vs 96.00%; *P* = .428). Kaplan–Meier analysis revealed consistent results, showing similar time-to-undetectable HBV DNA across treatment arms (Fig. 3; Table 3).

#### 3.3.2. Biochemical response

ALT normalization did not differ significantly at 6 months (57.69% vs 72.00%; *P* = .131) but showed a borderline trend favoring TAF at 12 months (73.08% vs 88.00%; *P* = .058). HBeAg seroconversion rates among baseline-positive patients did not differ significantly at either time point (both *P* > .05; Table 3).

### 3.4. Renal and bone safety and adverse events

TAF was associated with a markedly lower incidence of hypophosphatemia compared with TDF at both 6 months (0.00% vs 22.00%; *P* < .001) and 12 months (1.92% vs 28.00%; *P* < .001). New or worsening proteinuria occurred less frequently in the TAF group at 6 months (3.85% vs 16.00%; *P* = .039), and although the 12-month difference did not reach statistical significance (9.62% vs 18.00%; *P* = .219), the trend remained consistent.

Incidences of acute kidney injury, grade ≥ 3 AEs, and treatment discontinuation were low in both groups and did not differ significantly (all *P* > .05). These findings were directionally consistent in the PSM cohort (Table 4).

**Table 4:** Renal/bone safety and adverse events.

Outcome	TAF (n = 52)	TDF (n = 50)	*χ*²	*P*
Hypophosphatemia 0–6 mo, n (%)	0 (0.00%)	11 (22.00%)	12.823	<.001
Hypophosphatemia 0–12 mo, n (%)	1 (1.92%)	14 (28.00%)	13.819	<.001
AKI 0–6 mo, n (%)	2 (3.85%)	5 (10.00%)	1.510	.219
AKI 0–12 mo, n (%)	2 (3.85%)	7 (14.00%)	3.267	.071
New/worsened proteinuria 0–6 mo, n (%)	2 (3.85%)	8 (16.00%)	4.258	.039
New/worsened proteinuria 0–12 mo, n (%)	5 (9.62%)	9 (18.00%)	1.513	.219
Grade ≥ 3 adverse events 0–12 mo, n (%)	4 (7.69%)	2 (4.00%)	0.628	.428
Discontinuation due to AEs 0–12 mo, n (%)	1 (1.92%)	1 (2.00%)	0.001	.978

AEs = adverse events, AKI = acute kidney injury, TAF = tenofovir alafenamide, TDF = tenofovir disoproxil fumarate.

### 3.5. Subgroup and sensitivity analyses

**Figure 4.**
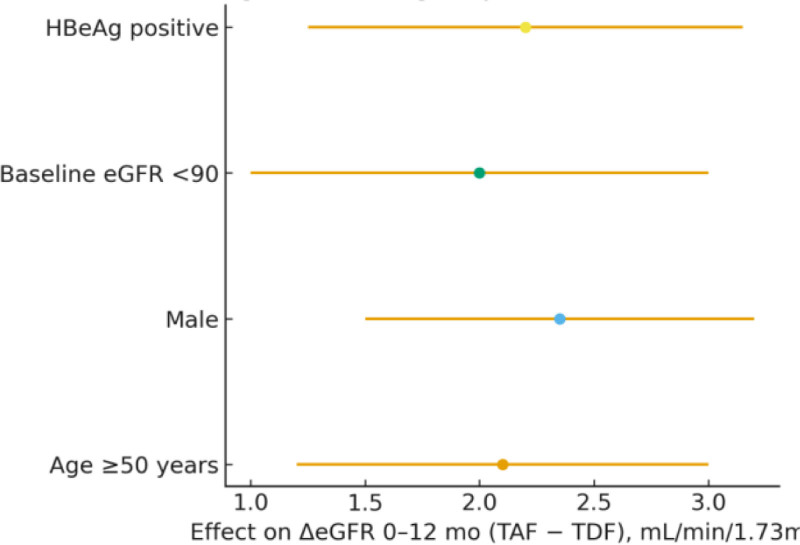
Subgroup forest plot for the primary endpoint (ΔeGFR 0–12 months). eGFR = estimated glomerular filtration rate.

Treatment effects on ΔeGFR at 12 months were consistent across predefined subgroups, including age (≥50 vs <50 years), sex, baseline eGFR (<90 vs ≥90 mL/min/1.73 m²), and HBeAg status (positive vs negative). No significant interactions were observed (interaction *P* = .151–.726), indicating stable treatment effects across clinical subgroups (Fig. 4; Table 5).

**Table 5:** Subgroup analysis for ΔeGFR 0–12 months (interaction *P*).

Subgroup (reference vs subgroup)	Effect in reference (ΔTAF−ΔTDF)	Effect in subgroup (ΔTAF−ΔTDF)	Interaction *P*
Age ≥ 50 yr	3.64	−0.45	.151
Male	3.80	2.31	.550
Baseline eGFR < 90	2.58	3.90	.620
HBeAg positive	3.10	2.31	.726

eGFR = estimated glomerular filtration rate, HBeAg = hepatitis B e antigen, TAF = tenofovir alafenamide, TDF = tenofovir disoproxil fumarate.

## 4. Discussion

This single-center retrospective cohort study compared the renal and bone safety and antiviral efficacy of TAF and TDF in treatment-naïve CHB patients within fixed follow-up intervals (6 and 12 months). The main findings were as follows: at 12 months, TAF demonstrated significantly better renal and bone safety, as reflected by higher ΔeGFR and lumbar spine BMD; HBV DNA undetectable rates were comparable between groups at both 6 and 12 months, while ALT normalization showed a borderline trend favoring TAF at 12 months; TAF significantly reduced the incidence of hypophosphatemia and showed less proteinuria at 0 to 6 months, with comparable rates of severe AEs and treatment discontinuation; and subgroup analyses revealed no significant interactions, suggesting consistent treatment effects across different populations. These findings align within the framework of “equivalent antiviral efficacy but improved renal and bone safety,” contributing additional short- to mid-term real-world evidence.

TAF, an optimized prodrug formulation, achieves equivalent antiviral potency while reducing plasma tenofovir exposure by approximately 90% compared with TDF, thereby decreasing the likelihood of proximal tubular and bone metabolism–related toxicity at the pharmacokinetic level. Consequently, with extended observation to 12 months, the advantages of TAF-greater eGFR stability, lower phosphate decline, and improved BMD-became more apparent, consistent with the gradual progression characteristics of renal and bone endpoints.^[8-12]^

Two pivotal phase III randomized, double-blind, non-inferiority trials in HBeAg-positive and HBeAg-negative CHB populations demonstrated that TAF was non-inferior to TDF in antiviral efficacy but superior in renal and bone safety, with these advantages persisting during prolonged treatment.^[8-10,12]^ Several real-world studies, including cohorts of patients switched from TDF to TAF, have consistently reported improvements in renal function parameters and BMD while maintaining viral suppression, supporting the consideration of therapy modification when renal or bone safety concerns arise.^[13-15]^ Our findings are consistent with these prior reports and add 2 novel aspects: first, the simultaneous quantification of renal, bone, and virological/biochemical outcomes using standardized platforms and consistent criteria; and second, the use of fixed 6- and 12-month observation windows, which better mirror typical clinical follow-up intervals.

For CHB patients with existing or potential renal and bone risks-such as older age, reduced baseline eGFR, low bone mass, or prior TDF-related renal/bone adverse signals-TAF may represent a preferable or earlier therapeutic choice. While maintaining comparable virological suppression, TAF may reduce events such as hypophosphatemia and proteinuria, thereby enhancing long-term safety and adherence. International guidelines likewise emphasize individualized regimen selection and dynamic monitoring of renal and bone health (eGFR, serum phosphate, urinary protein, and DXA as indicated), consistent with our findings.^[16]^ It should also be noted that several studies have reported potential metabolic effects following a switch from TDF to TAF, including increased serum lipids and body weight, highlighting the need for metabolic monitoring and management, particularly in patients with cardiometabolic risk factors. Balancing renal and bone benefits with metabolic considerations offers a more realistic and individualized approach to clinical management.^[17,18]^

Strengths and limitations: This study has several strengths. The use of predefined follow-up windows and standardized measurement platforms minimized information and measurement heterogeneity. The concurrent presentation of renal, bone, and virological outcomes improved clinical interpretability. The application of PSM enhanced comparability, and Fisher’s exact test ensured robustness for rare events. However, several limitations should be acknowledged: The single-center design and exclusion of patients with severe renal impairment or metabolic bone disease limit the generalizability of our findings to broader CHB populations with more diverse comorbid profiles; DXA assessments and follow-up were limited to 12 months, and longer-term changes in bone mass or hard outcomes remain to be validated; the single-center, relatively small sample size may limit generalizability; and no formal adjustment for multiple comparisons was applied, and results were interpreted based on effect sizes and 95% CIs. In addition to measured variables, unmeasured confounders such as vitamin D intake, physical activity, and concurrent medications may have influenced renal and bone outcomes. Although sensitivity analyses like *E*-values could quantify such potential bias, these were not feasible due to limited sample size and outcome frequency. These limitations are consistent with prior real-world evidence and underscore the need for larger, prospective, and longer-term studies to verify these findings.^[19]^

## 5. Conclusion

TAF showed superior renal and bone safety compared with TDF after 12 months of treatment, reflected by improved ΔeGFR and lumbar spine BMD, while maintaining comparable antiviral efficacy. TAF also reduced hypophosphatemia and early proteinuria without increasing severe AEs or discontinuations. These findings indicate that TAF is a safer and more sustainable long-term option for CHB patients with renal or bone risks. Further multicenter studies with longer follow-up are needed to confirm its long-term renal, bone, and metabolic outcomes.

## Author contributions

**Conceptualization:** Lizhi Wang, Guotao Pi, Dan Lu, Yan Wang, Wenjin Sun.

**Data curation:** Lizhi Wang, Guotao Pi, Dan Lu, Yan Wang, Wenjin Sun.

**Formal analysis:** Lizhi Wang, Guotao Pi, Dan Lu, Yan Wang, Wenjin Sun.

**Funding acquisition:** Lizhi Wang, Guotao Pi, Dan Lu, Yan Wang, Wenjin Sun.

**Investigation:** Guotao Pi, Wenjin Sun.

**Writing – original draft:** Lizhi Wang, Dan Lu, Wenjin Sun.

**Writing – review & editing:** Lizhi Wang, Dan Lu, Wenjin Sun.
